# Emergency Response to Australia’s Black Summer 2019–2020: The Role of a Zoo-Based Conservation Organisation in Wildlife Triage, Rescue, and Resilience for the Future

**DOI:** 10.3390/ani11061515

**Published:** 2021-05-23

**Authors:** Marissa L. Parrott, Leanne V. Wicker, Amanda Lamont, Chris Banks, Michelle Lang, Michael Lynch, Bonnie McMeekin, Kimberly A. Miller, Fiona Ryan, Katherine E. Selwood, Sally L. Sherwen, Craig Whiteford

**Affiliations:** 1 Wildlife Conservation and Science, Zoos Victoria, Parkville, VIC 3052, Australia; lwicker@zoo.org.au (L.V.W.); alamont@zoo.org.au (A.L.); cbanks@zoo.org.au (C.B.); fryan@zoo.org.au (F.R.); kselwood@zoo.org.au (K.E.S.); ssherwen@zoo.org.au (S.L.S.); cwhiteford@zoo.org.au (C.W.); 2 Healesville Sanctuary, Badger Creek, VIC 3777, Australia; kmiller@zoo.org.au; 3 Marketing, Communications & Digital Strategy, Zoos Victoria, Parkville, VIC 3052, Australia; mlang@zoo.org.au; 4 Melbourne Zoo, Parkville, VIC 3052, Australia; mlynch@zoo.org.au; 5 Werribee Open Range Zoo, Werribee, VIC 3030, Australia; bmcmeekin@zoo.org.au

**Keywords:** wildlife, bushfires, emergency response, conservation, animal welfare, threatened species, endangered species, catastrophic events, disasters, resilience

## Abstract

**Simple Summary:**

In the summer of 2019–2020, a series of more than 15,000 bushfires raged across Australia in a catastrophic event called Australia’s Black Summer. An estimated 3 billion native animals, and whole ecosystems, were impacted by the bushfires, with many endangered species pushed closer to extinction. Zoos Victoria was part of a state-led bushfire response to assist wildlife, alongside government, non-government organisations, and key partners. Here, we detail the role of Zoos Victoria in wildlife triage and welfare, threatened species evacuation and recovery, media and communications, and fundraising during and after the fires. We provide case studies on the triage, care, release, and monitoring of injured koalas (*Phascolarctos cinereus*); the evacuation and return of endangered eastern bristlebirds (*Dasyornis brachypterus*) and brush-tailed rock wallabies (*Petrogale penicillata*); and the development of nutritionally suitable supplementary food and emergency feeding of critically endangered mountain pygmy-possums (*Burramys parvus*). We share our strategies for future resilience and readiness for similar catastrophic events, as well as the development of triage protocols, emergency response kits, emergency enclosures, captive breeding programs, and nature-based healing for communities directly affected by the fires. We hope that by outlining these contributions from a zoo-based conservation organisation, other zoos and wildlife organisations, both nationally and internationally, may be assisted or encouraged to commit resources and build expertise to assist wildlife in catastrophic events.

**Abstract:**

Modern zoos are increasingly taking a leading role in emergency management and wildlife recovery. In the face of climate change and the predicted increase in frequency and magnitude of catastrophic events, zoos provide specialised expertise to assist wildlife welfare and endangered species recovery. In the 2019–2020 Australian bushfire season, now called Australia’s Black Summer, a state government-directed response was developed, assembling specialised individuals and organisations from government, non-government organisations, research institutions, and others. Here, we detail the role of Zoos Victoria staff in wildlife triage and welfare, threatened species evacuation and recovery, media and communications, and fundraising during and after the fires. We share strategies for future resilience, readiness, and the ability to mobilise quickly in catastrophic events. The development of triage protocols, emergency response kits, emergency enclosures, and expanded and new captive breeding programs is underway, as are programs for care of staff mental health and nature-based community healing for people directly affected by the fires. We hope this account of our response to one of the greatest recent threats to Australia’s biodiversity, and steps to prepare for the future will assist other zoos and wildlife organisations around the world in preparations to help wildlife before, during, and after catastrophic events.

## 1. Introduction

### 1.1. The Role of Zoos in Emergencies

Worldwide, zoos are increasingly responding to emergencies and catastrophic events that affect wildlife, including bushfires, floods, cyclones, and oil spills, as well as more individual welfare cases such as entanglements, road trauma, and disease [1]. Specialised zoo emergency teams (which include zookeepers, managers, scientists, emergency coordinators, veterinarians, and others) and zoo-based veterinary hospitals are taking a leading role in crisis management and wildlife recovery. With an ever-expanding human population, increasing issues of deforestation and pollution [2], and the compounding effects of climate change [3], localised and widespread emergencies are becoming more prevalent and increasing in both frequency and magnitude [4]. Climate change in particular is predicted to exacerbate catastrophic bushfire conditions. By 2050 in Australia, days with high-to-extreme fire risk are forecast to increase by 15–70% compared with 2010. By 2100, it is predicted that this increase will be greater than 100% [5].

Zoos Victoria, a not-for-profit zoo-based conservation organisation in Victoria, Australia, has a long-term commitment to wildlife welfare and threatened species conservation. In particular, we have priority programs to aid the recovery of 27 endangered South Eastern Australian species (based on the International Union of the Conservation of Nature (IUCN) Red List criteria, known as Zoos Victoria’s ‘Fighting Extinction’ species), as well as an additional eight Watch List species that are in need of further attention [6]. We run wildlife hospitals at three properties: Healesville Sanctuary, Melbourne Zoo, and Werribee Open Range Zoo, with the Australian Wildlife Health Centre at Healesville Sanctuary treating over 1500 cases of sick and injured wildlife per year. Key drivers for wildlife injury vary, but often include trauma and death from motor vehicle collisions, for example in echidnas (*Tachyglossus aculeatus*) [7] and reptiles [8]; entanglement in discarded waste, fruit tree netting, and barbed-wire fencing, for example in fruit bats (*Pteropus poliocephalus*) [9]; and attacks by domestic dogs (*Canis familiaris*) and cats (*Felis catus*) on a range of taxa (Zoos Victoria unpublished data). Further threats include heat stress and burns following bushfires and during heat wave events, infectious disease, and toxicities such as rodenticides or ingestion of agricultural and industrial chemicals. A specialised Marine Response Unit at Melbourne Zoo treated more than 470 cases of injury, entanglement, and illness in marine species, including seals and water birds, in 2019 alone. Trained wildlife and veterinary teams work alongside government and non-government organisations (NGOs) and the volunteer wildlife care community to respond to wildlife emergencies throughout the year.

In February 2009, Zoos Victoria was central to the wildlife response following the Black Saturday bushfires, treating burnt and heat-stressed reptiles, echidnas, koalas (*Phascolarctos cinereus*), eastern grey kangaroos (*Macropus giganteus*), and other species. At the height of those fires, we also successfully evacuated Healesville Sanctuary, including the animals, for the first time in its 86-year history. Many changes were made state-wide to policy and planning after those fires [10]. Following Black Saturday, Zoos Victoria instigated new twice-yearly evacuation training and purchased new emergency kits, transport equipment, and housing for animals in our care. Despite our long-term management of wildlife and experiences with some of the worst bushfires in Victoria’s history, we were still shocked by what we experienced in the summer of 2019–2020—a catastrophic series of fires colloquially named the Black Summer.

### 1.2. Australia’s Black Summer 2019–2020

From September 2019 to March 2020, a series of more than 15,000 bushfires raged across Australia, which peaked at the height of summer in December 2019 and January 2020 [11]. While fire forecasters had predicted a potentially catastrophic fire season due to long-term drought, dry conditions, and high forecasted temperatures, the fires were unprecedented in their size and impacts on biodiversity and animal welfare. The fires killed 33 people, destroyed 5900 buildings, and burnt an estimated 18.6 million hectares (an area larger than France), much of which was native bushland. While fires affected all Australian states and territories, a State of Emergency was declared for New South Wales (NSW) in November 2019, and in the Australian Capital Territory (ACT) in January 2020. A State of Disaster was declared in Victoria in January 2020; the first time such powers had been used since they were added to the Emergency Management Act 1986, following the 2009 Victorian bushfires [12]. The Black Summer bushfires impacted an estimated 3 billion native animals, with many endangered species pushed closer to extinction.

An estimated 2.46 billion of the affected animals were reptiles, with amphibians, birds, and mammals comprising the remaining victims [13]. The number of animals impacted by the bushfires is likely to be far greater, as the estimates do not include aquatic species or invertebrates. While many animals were killed by the fires, the ongoing effects of lack of water, food, and shelter; stress; and exposure to predators, including introduced species such as feral cats and red foxes (*Vulpes vulpes*), led and will continue to lead to greater mortalities. The toxic sludge of ash and debris that washed into creek and river systems added an additional ongoing threat to terrestrial and aquatic species [14].

Australia’s unique and complex ecosystems are under increasing strain. Changes in land-use, natural hazards, habitat loss and degradation, and feral animal and invasive plant species are contributing to increasingly poor ecological prospects, with the impacts of climate change exacerbating existing pressures. Scientists have predicted serious, long-term, adverse effects on biodiversity due to the Black Summer. Over 330 threatened species and 37 threatened ecological communities were in the path of the bushfires. Species and communities not previously listed as threatened under national environmental law may now be threatened as a consequence of the Black Summer bushfires [11].

In late 2019, Zoos Victoria was called on to work alongside partners in southeastern Australia to assist with the unfolding threat to wildlife, as well as the threat to biodiversity at a national scale. Strong existing and new partnerships and alliances with other organisations, including government departments, researchers, NGOs, other zoos, and species experts were paramount in developing and managing the emergency response during and after the fires. This paper details Zoos Victoria’s role in emergency management, governance, veterinary triage, rescue, and release of native wildlife; supplementary feeding; stakeholder engagement; public communication; and fundraising response to the Black Summer. It details key learnings, outcomes, and strategies to prepare for future catastrophic events and to promote the resilience and recovery of native species and their habitats.

## 2. Zoos Victoria’s Black Summer Response

### 2.1. Emergency Governance

The Victorian Department of Environment, Land, Water, and Planning (DELWP) has responsibility for the state’s wildlife and leads the official response to the wildlife health and welfare impacts of environmental emergencies, including bushfires. However, the sheer scale of the fires of 2019–2020 required a response that was larger and broader than ever before. Consequently, the role of Zoos Victoria in the state-led response was also substantially greater than in previous emergencies.

**Figure 1:**
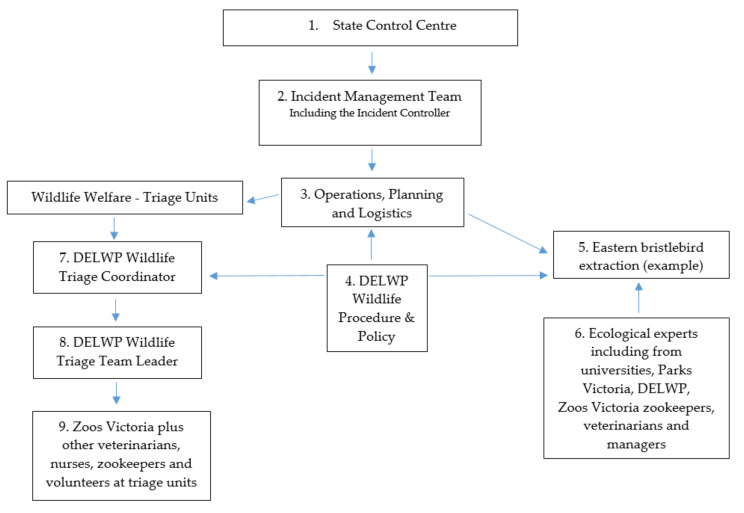
A simplified flow chart of Zoos Victoria’s involvement in the state-led emergency response, including wildlife triage governance and the eastern bristlebird extraction as examples. Numbers in the figure boxes correspond to the numbered explanations below. The structure includes: (**1**) State Control Centre (SCC), which oversees all emergencies in the state applying an Australasian Inter-Service Incident Management System (AIIMS), and comprises personnel from state agencies, including Emergency Management Victoria, Country Fire Authority (CFA), Department of Environment, Land, Water and Planning (DELWP), Parks Victoria, Victoria Police, and State Emergency Service amongst others. In the 2019–2020 bushfires, a State Controller Wildlife was included in the SCC. (**2**) The Incident Management Team (IMT), led by an Incident Controller, manages a specific emergency close to or near the emergency under an AIIMS configuration, and is responsible for all activities undertaken in the emergency response. (**3**) Operations, planning, and logistics are three of the groups within the AIIMS structure assisting wildlife. (**4**) All activities are undertaken in accordance with DELWP Wildlife Procedures and Policies, including operations, planning, and logistics, and the case studies are shown here. (**5**) The eastern bristlebird extraction, as an example, was authorised and conducted under the auspices of the IMT, which also supported aspects including operational planning and transport logistics. (**6**) The bristlebird extraction team comprised people from multiple organisations with expertise in ecology, animal health, handling, and keeping. Melbourne Zoo and Healesville Sanctuary zookeepers, veterinarians, and management cared for the birds ex situ at those locations. (**7**,**8**) Triage units are one of the operational units within the structure, with a DELWP Wildlife Triage Coordinator overseeing the triage efforts and a DELWP Triage Team Leader at each site. (**9**) Triage units are staffed from multiple organisations with experience in wildlife assessment and clinical management, with additional support and training provided by Zoos Victoria staff.

In early January 2020, DELWP invited collaboration from Victoria’s environment sector to respond to the growing bushfires. Zoos Victoria was asked to lead programs extracting threatened terrestrial animal species identified as at risk and to participate in a forum to develop a comprehensive Bushfire Biodiversity Response and Recovery Plan (BBRRP) [15]. This forum saw participants from conservation NGOs; specialists from universities and research centres; and state agencies such as Zoos Victoria, the Royal Botanic Gardens (RBG; Melbourne and Sydney, Australia), and Taronga Conservation Society (Taronga CS) develop a plan and present it to the State’s Environment Minister on the day. The Minister responded by committing over AUD17 million as a down payment to allow the plan’s immediate implementation. This was an unprecedented financial commitment to wildlife and the environment affected by bushfires in Victoria. A second forum in late February 2020 consolidated the BBRRP. Zoos Victoria’s role was to co-lead and act in three of the seven themes in the plan (see Appendix A). Additionally, a Zoos Victoria Director was embedded in the Victorian State Control Centre (SCC; the emergency response-coordinating entity) to assist and advise in the high-level coordination of wildlife protection and welfare within these themes (see Figure 1 for a simplified flow chart of the wildlife elements of the state-led emergency response).

A series of national initiatives were instigated as the scale of the bushfire emergency became apparent. The Australian Threatened Species Commissioner, at the request of the Australian Minister for the Environment, formed a Wildlife and Threatened Species Bushfire Recovery Expert Panel to help ensure a national coordinated response to the growing threat to Australia’s biodiversity. Zoos Victoria’s Chief Executive Officer contributed expertise in ex situ conservation in recognition of the increasingly significant role zoos are playing in the care, protection, and conservation of rare and endangered species. This group identified 119 species, whose vulnerability was exacerbated by the fires, as requiring attention. Following the fires, this helped direct Australian Commonwealth government funding to areas and species of greatest need. The Australasian Zoo and Aquarium Association (ZAA) conducted a prioritisation workshop for species that would benefit from ex situ conservation, and funding was provided to zoos and sanctuaries around Australia to support the ex situ conservation of 12 threatened species.

In NSW and South Australia (SA), zoo-based conservation organisations, Taronga CS and Zoos South Australia (Zoos SA), assisted the wildlife response in their respective states. Weekly briefings were convened across Taronga CS, Zoos SA, and Zoos Victoria during the height of the fires to provide support to one another and facilitate consistency of messaging around the capability of Australia’s zoos in responding to the bushfire emergency.

Following the fires, the DELWP convened two further forums: one for wildlife welfare and a second for broader environmental recovery. Zoos Victoria is involved in, or in the case of amphibians, leading the DELWP specialist taxon group assessments to investigate the status of species and recovery actions needed to promote future resilience. A multi-state New Holland mouse (*Pseudomys novaehollandiae*) two-day workshop was held by Zoos Victoria (facilitated remotely due to a COVID-19 lockdown in Victoria) in February 2021, with plans for a smoky mouse (*P. fumeus*) workshop at Melbourne Zoo under development. Zoos Victoria’s contribution within the seven DELWP-led bushfire themes are as follows below.

### 2.2. Theme 2—Wildlife Welfare

During the Black Summer, immediate emergency response was required in the veterinary assessment and care of native wildlife impacted by the fires. Under the guidance of DELWP procedures and policies, wildlife in areas directly affected by, or close to, bushfires were initially assessed in the field by trained assessment teams. Animals were euthanased immediately on humane grounds if this was warranted due to the severity of their injuries; left in place if the habitat was considered safe with adequate carrying capacity; translocated by DELWP wildlife officers to nearby unburnt habitat (following processes prescribed in Victoria’s Koala Management Strategy [16]); or transferred to triage units for further assessment by wildlife veterinary teams. Zoos Victoria’s veterinary teams, in collaboration with other veterinary partners including the Australian Veterinary Association (AVA), worked alongside DELWP to establish four emergency triage units under the management of the regional Incident Management Team (IMT), which sits under the SCC (Figure 1). In Victoria, triage units were established at a local community hall in Mallacoota (Mud Hut Pavilion), a church hall in Corryong, and as part of a formal partnership with the Royal Society for the Prevention of Cruelty to Animals (RSPCA) Victoria, in a purpose-built mobile veterinary truck, the RSPCA MAC (Mobile Animal Care Unit) at Bairnsdale and Gelantipy [17]. Of close to 3000 animals assessed in the field, 259 were sent to wildlife triage units for full assessment under anaesthesia by wildlife veterinary teams (unpublished data, Zoos Victoria and DELWP). The bushfires were so intense and widespread that billions of animals perished in the flames, leaving a relatively small number of survivors encountered.

A DELWP Wildlife Triage Coordinator oversaw the operations of the triage units and DELWP Triage Team Leaders oversaw daily management and logistics support of the triage unit. Wildlife-experienced veterinarians and veterinary nurses from Zoos Victoria, Ballarat Wildlife Park, and Taronga CS provided the mainstay of veterinary expertise, with husbandry support by experienced zookeepers on site. The AVA, RSPCA Victoria, the University of Melbourne, Vets for Compassion, and other independent, experienced veterinarians also formed part of the coordinated response. Zoos Victoria developed and collated 10 triage unit response kits with support from the AVA at the request of DELWP. Each kit contained all necessary equipment, consumables, and drugs to establish a triage unit ‘field clinic’ for rapid deployment to treat injured and fire-affected native wildlife. Each kit contained examination tables, temporary animal enclosures, anaesthetic equipment, veterinary consumables necessary for managing burns, drugs, intravenous fluid therapy supplies, supplementary feeding equipment, and stationery supplies to ensure teams were well prepared. Kits were packed into tough tubs for transport, with each containing instructions for triage staff in set up and in-triage operations, ensuring streamlined systems for uninterrupted operations during the response. The development of the triage kits has provided an invaluable resource and ensures preparedness to respond at short notice to events where wildlife has been impacted.

While most of the animals presenting to triage units were koalas (75%, unpublished data, Zoos Victoria and DELWP), a variety of species were seen, including the tiny feathertail glider (*Acrobates pygmaeus*) (1.9%, unpublished data, Zoos Victoria and DELWP), grey-headed flying fox (2.7%, unpublished data, Zoos Victoria and DELWP), and eastern grey kangaroo (5%, unpublished data, Zoos Victoria and DELWP), with the remaining 15.4% made up of 34 species, including the superb lyrebird (*Menura novaehollandiae*), tawny frogmouth (*Podargus strigoides*), lace monitor (*Varanus varius*), and a red-bellied black snake (*Pseudechis porphyriacus*). The prognosis for successful treatment and rehabilitation was carefully assessed considering the location and severity of burns and other fire-related injuries (including smoke inhalation, heat stress, and dehydration), other health issues or injuries (including fractures or bite wounds), and the age and body condition of the animal. As a result, 20% of animals presented to triage units were euthanased after initial veterinary examination. However, close to half (42%) were released back to the wild within 24 h of presentation, following emergency first aid and supportive care, such as intravenous fluid therapy to correct dehydration, treatment for minor wounds, and for koalas, feeding with a special critical care paste made by blending eucalyptus leaves in water. These animals were released either directly back to their original location or as close to it as possible, as outlined in Victoria’s Wildlife Rehabilitation Authorization Guide (VWRAG) [18]). Some 35% of animals presenting to triage required hospitalisation or ongoing care for greater than 24 h. Zoos Victoria veterinarians transferred a smaller number of koalas into longer-term care. These included six juveniles that were uninjured but orphaned, who were subsequently hand-raised by DELWP registered wildlife carers. Twenty-seven more severely affected koalas required hospitalisation and ongoing veterinary care not possible in a field triage unit. These koalas were transferred to purpose-built wildlife hospitals at Melbourne Zoo and Healesville Sanctuary, with some of them evacuated with veterinary teams on airplanes via the Royal Australian Air Force.

#### 2.2.1. Case Study—Veterinary Care, Rehabilitation, and Return of East Gippsland’s Koalas

The koalas discussed above that were initially stabilised in triage units before being transferred out of fire zones were critically injured. However, Zoos Victoria veterinarians assessed these animals as having a good prognosis for release back into the wild, provided they were able to receive the level of intensive, specialised veterinary and husbandry care available at both Healesville Sanctuary and Melbourne Zoo. The koalas underwent regular burn debridement and wound care under general anaesthesia to manage their wounds and promote healing. This period of intensive care of burns was completed within three weeks in the majority of animals, but some individuals with the most severe injuries required procedures for up to eight weeks. Pain was proactively and intensively managed; high-quality, specialized nutrition was provided to meet increased energy needs for healing and koala-specific hospital enclosures were provided, which allowed the animals space to move and forage in quiet surroundings. Experienced veterinary nurses and zookeepers closely monitored their behaviour, appetite, and faecal output. Health status and response to treatment were closely monitored using diagnostic pathology tests, including blood bio-chemistry and haematology assessments; and imaging, including radiographs, magnetic resonance imaging (MRI), and detailed ultrasonography. Once burns and other fire-related injuries had healed and the koalas were assessed by veterinary staff as no longer requiring intensive veterinary management, they were transferred to large, purpose-built pre-release enclosures at Healesville Sanctuary and to partners at Phillip Island Nature Parks (PINP) for their next phase of rehabilitation.

While in these naturalistic enclosures, koalas were given the space and time to recuperate mentally, while building the climbing strength, fitness, and vitality required to survive release to the wild. Behaviour, dietary intake, and faecal output were closely monitored, with regular updates provided to veterinarians to ensure all koalas were displaying health and behaviours appropriate for successful integration into wild populations. In October 2020, koalas in care at Healesville Sanctuary and PINP, as well as hand-raised orphans in the care of registered wildlife carers, received a detailed pre-release health assessment under general anaesthesia, including disease screening for *Chlamydia* spp. and *Herpesvirus*, assessment of kidney function, and weight and body condition scoring. Following this, 14 koalas were fitted with radiotelemetry and global positioning system (GPS) collars (Lotek Lite Track 60 RF GPS Pinpoint collar, Lotek, New Zealand), and between November and December 2020, close to 11 months after they had first presented during the fires, were released into regenerating bushland in East Gippsland, as close as possible to their origin. Release locations were determined after consideration of VWRAG [18] and the translocation site criteria outlined in the state’s Koala Management Strategy [16], and included an assessment of habitat suitability for koalas, as well as the long-term sustainability of pre-existing koala populations already resident at the site.

As part of a collaborative project with Zoos Victoria, DELWP, Parks Victoria, Federation University, ecologists from EcoPlan Australia, and local communities who live near the release locations, we are now carefully monitoring koalas to better understand their health, habitat use, and behaviour following release to the wild (see monitoring section below). Non-invasive faecal samples allow greater understanding of the genetics and disease status of the resident koalas at release locations and will enable the team to monitor released koalas into the future. Information gained from this unique project will help wildlife agencies to better understand the welfare implications of the release of rehabilitated, fire-affected koalas (and other species); provide information to enable evaluation of existing veterinary care, rehabilitation, and pre-release assessment protocols; and will result in significantly better decision making during wildlife emergency responses in the future.

#### 2.2.2. Supplementary Food and Water

Following habitat destruction by bushfires, the provision of food and water to surviving animals is often suggested, however this comes with inherent risks [19]. The area must be surveyed to determine which species survived and in what number; the extent of the fire and impacts on vegetation and clean water; the distance to the nearest suitable food and water source; and other key threats. Only nutritionally suitable foods should be provided, which must be in a hygienic and safe manner for any species present. The area needs to be monitored for safety and efficacy of the feed stations, as well as risk of threats such as gastrointestinal illness, aggression, or predation. There must be a planned exit strategy for the reduction of supplementary food as natural foods become available. Care must be taken to not negatively affect the burnt area, as weeds (e.g., from the provision of unsterilized hay) can outcompete regenerating native flora [20]. The safety of staff and volunteers in the area must be considered and monitored during food provisioning. Following the Black Summer fires, Zoos Victoria collated tables of suitable food and availability, plus other key considerations, threats, and risks for DELWP to consider regarding the feeding of threatened, and more commonly, fire-affected native species. For critically endangered species in particular, food shortages can preclude recovery or even result in local extinctions. Zoos Victoria staff later joined other members of the Food and Water Working Group of Wildlife Health Australia (WHA) to develop national guidelines for supplementary food and water during and after the fires [20]. We provided advice and food and water stations to assist key macropod species, such as brush-tailed rock wallabies (*Petrogale penicillata*), with DELWP, Parks Victoria, and Wildlife Victoria managing staff and volunteers keen to assist wildlife. Zoos Victoria staff members continue to contribute to this work as members of the ongoing WHA supplementary feeding subgroup to refine guidelines and public messaging in preparation for future emergencies or catastrophic events.

#### 2.2.3. Case Study—Feeding Mountain Pygmy-Possums Nutritionally Suitable ‘Bogong Bikkies’ after the Fires

In spring 2017 and 2018, the billions of Bogong moths (*Agrotis infusa*) that migrate annually to alpine areas of Victoria and NSW largely failed to arrive. Bogong moths are the key food source for the critically endangered mountain pygmy-possum (*Burramys parvus*; MPP) during their spring–summer breeding period. In all Victorian monitored MPP populations, 50–95% of females lost their litters of pouch young by the end of December 2018 [21]. The mothers were in poor body condition, often no longer lactating, and post-mortem examination of pouch young indicated that starvation was the most likely cause of death (Zoos Victoria, unpublished data). In 2019, as part of a state-wide MPP contingency plan [21], Zoos Victoria, working with world experts in veterinary nutrition, including Professor Ellen Dierenfeld (Nottingham Trent University) and Dr Michelle Shaw (Taronga CS), developed a novel, shelf-stable supplementary biscuit-like food source, named ‘Bogong Bikkies’. The formulation was based on our previous analysis of the macro- and micro-nutrient analysis of the Bogong moth and was commercially developed as a human-grade food product through Zoos Victoria’s partners, Wombaroo (Wombaroo Passwell, Australia) [19]. The food and various types of feeders were successfully tested with MPPs in our care at Healesville Sanctuary for safety, efficacy, palatability, and stability. Working with Parks Victoria, we then trialed the food and three types of feeders under field conditions from November 2019 to April 2020. This work had to be halted for two weeks during the height of the nearby Black Summer fires due to dangerous fire conditions. However, it was then able to continue in the publicly closed and evacuated alpine parks under special permission from emergency services, following additional safety measures (including pre-field fire meetings and frequent radio safety check-ins). The MPPs and other small mammals readily consumed the supplementary food. We saw no changes in predator activity in the area and no evidence of intra- or inter-specific competition around the supplementary food stations.

While we developed this food in response to the collapse in Bogong moth numbers, this forethought proved crucial following the Black Summer fires. In NSW, populations of MPPs in northern Mt Kosciusko National Park were badly affected by fires, leaving no food or vegetation for surviving MPPs and other local species. Zoos Victoria was able to package the ‘Bogong Bikkies’ and the most successful feeder prototypes to send to our partners in the NSW Department of Planning, Industry, and Environment’s (DPIE) Saving our Species team, for their wide-scale, intensive program to feed the MPPs, as well as other surviving species (including native rodents, marsupials, and lizards) impacted by the fires [19]. Animals readily consumed the food and gained weight prior to their hibernation period (DPIE pers comm). This case study highlights a model for, and the importance of, a proactive approach to protection and recovery of species following emergencies.

### 2.3. Theme 3—Threatened Species Extractions

Zoos Victoria, in conjunction with DELWP and Melbourne’s RBG, co-led the activities in Theme 3 [15]. The Arthur Rylah Institute (ARI), DELWP’s biological research arm, led the extraction of threatened freshwater animals from fire-affected waterways (see similar examples following the 2009 Black Saturday fires in Hames [22]). The RBG led the extraction of plants and fungi from fire areas in Victoria, and Zoos Victoria was asked to lead the rescue of terrestrial animals where required. In December 2019, as fires spread across NSW and continued to grow in Victoria, Zoos Victoria began collating the emergency holding capacity, dietary requirements, and transport box availability across our three zoos, in case rescue and housing became necessary. This included quarantine considerations; availability of appropriate veterinary resources (including sufficient experienced veterinary staff, hospital and rehabilitation housing, and the ability to provide sufficient food for the expected number of animals during the intensive care and rehabilitation phases); species-specific holding facilities (for those species requiring specific temperatures, aquatic housing, or other key considerations); and enclosures that could house a variety of species depending on the areas of fire impact. Across the three zoos, staff reviewed and prepared enclosures, food stores, and built new transport boxes and holding areas. As the fires grew in size and intensity, Zoos Victoria employed additional staff to conduct a regional institutional capacity audit to determine enclosure and housing ability with partners across Victoria, including other zoos, wildlife parks, and government partners, such as Parks Victoria and PINP. A comprehensive document detailing enclosure, transport box, and equipment capacities now exists to prepare for future rescues of threatened and other species where needed. Prior to each bushfire season, we will ensure our preparedness by confirming institutional contacts, capacity, and willingness to support wildlife rescue work.

#### 2.3.1. Case Study—The Rescue and Release of the Endangered Eastern Bristlebird

In February 2020, as fires threatened Cape Howe in Victoria, growing concern was raised for a tiny endangered bird, the eastern bristlebird (*Dasyornis brachypterus*). With fewer than 2500 birds remaining in Australia, and around 400 of these in a population across the Victorian–NSW border, losing these animals would be a massive blow to the survival of the species. Following an alert from ARI fire modelers, in a novel operation, Zoos Victoria worked with DELWP, a team of specialists from Monash and Wollongong Universities, and government agencies to retrieve up to 20 eastern bristlebirds from Howe Flat, Victoria, ahead of the fire front and transport them to Melbourne Zoo for safekeeping. The birds would serve as founders for a captive breeding population should their habitat be destroyed [23]. Due to increasing fire threat, the operation was called short after a few days and the team was evacuated. However, in that time the team was able to successfully collect 15 birds and transport them to Melbourne Zoo. As Zoos Victoria had not held this species previously, advice from partners at Monash and Wollongong Universities and Currumbin Wildlife Sanctuary was crucial to the birds’ ex situ management.

Despite best-practice care provided by experienced keeping and veterinary staff, and considerable planning and efforts to mitigate the stress of capture, transport, and captivity, six of the birds died due to the stress-induced respiratory illness, aspergillosis, in their initial days in captivity. Aspergillosis is an infectious, non-contagious disease caused by the globally ubiquitous, saprophytic fungi *Aspergillus* spp. [24]. Reported for a wide range of vertebrates, including humans, aspergillosis is a known health risk in the translocation of threatened birds [25]. *Aspergillus* spp. act opportunistically as pathogens in immunocompromised hosts, with infection in humans and animals most commonly occurring via inhalation of spores from the environment. Once clinical signs are shown in birds, mortality rates are high [26]. Birds were not initially placed on prophylactic treatment for *Aspergillus* spp. as it was judged that the stress of catching the birds in order to reliably medicate them would outweigh the benefits of medication. However, following the diagnosis of aspergillosis on necropsy investigation (gross and histological evaluation of tissues), the remaining birds were treated prophylactically, as best as possible, by medicating food items. This, and likely reduced stress following acclimatisation to their new environment, prevented further mortality. The zoonotic potential of the *Aspergillus* infections in the birds was assessed and judged to be insignificant for staff managing these animals. This was based on the ubiquitous nature of this fungus in the environment and assessment that the birds would be shedding negligible spores. One bird had sustained a broken leg during capture, so Zoos Victoria provided veterinary care at Healesville Sanctuary. While the fracture repair was successful, residual nerve damage left it unfit for release, thus it has been retained in care. Fortunately, the eastern bristlebird habitat at Howe Flat did not burn. We cared for the remaining birds at Melbourne Zoo and prepared them for release.

Despite looming restrictions due to the global COVID-19 pandemic, a flock of seven birds was returned to Howe Flat in April 2020. Another bird, which was unwell in April, was returned in October that year. An operation that would normally take months was planned and executed in less than a week [23]. This was possible due to an existing proposed translocation plan developed by DELWP; availability of expertise from universities, DELWP, and Parks Victoria to work alongside Zoos Victoria staff; the ready issuing of emergency permits to collect and move birds; and an exemption from the Environment Protection and Biodiversity Conservation Act 1999 (EPBC Act) to collect the animals. Another critical aspect was the logistics arrangements undertaken by the IMT (see Figure 1), which saw Australian and Singaporean Defence Force aircraft deployed to transport teams and birds to and from Melbourne, and Victorian Fisheries Authority vessels available to transport staff and birds across the adjacent Mallacoota estuary. There was also a considerable effort by zookeepers at Melbourne Zoo to modify empty eastern barred bandicoot (*Perameles gunnii*) enclosures to receive the birds. Fortuitously, the enclosures were available due to a large recent release of bandicoots to French Island, Victoria [27]. While the Victorian endangered eastern bristlebird habitat was spared and birds were able to be released back to their original locations, fires tore through the habitat in NSW. Many of the 250 birds in that habitat are presumed dead, underlining the importance of the rescue and work to help this species [23].

We have incorporated the experiences gained during this extraction event into Zoos Victoria response plans so that disease risk mitigation steps are pre-planned, and future extractions will result in better health and welfare outcomes for wildlife threatened by environmental emergencies. This rescue and release program can serve as a baseline from which improved models for future interventions can be developed to prevent extinction of species during bushfires and other catastrophic events.

#### 2.3.2. Case Study—Evacuating Brush-Tailed Rock Wallabies from the ACT

It is not only wild animals that may need to be evacuated (as above), but also animals from zoos and sanctuaries under bushfire threat. The critically endangered southern brush-tailed rock wallaby is one of Zoos Victoria’s 27 Fighting Extinction species. We work with partners at Tidbinbilla Nature Reserve (TNR) in the ACT to recover this species, and are supporting their new free-range, introduced-predator-free wallaby enclosure. Fires threatened TNR in January 2020 and emergency enclosures at Healesville Sanctuary were prepared, including work by horticulture, operations, zookeeper, and veterinary teams. Zoos Victoria instated new biosecurity measures and zookeepers had a new temporary quarantine changing room retrofitted nearby to separate the wallabies from other species on site.

The evacuation to Healesville was not needed in January, however in February the bushfire threatened TNR from a different direction and evacuation was necessary. Due to the prior preparation, we were ready to receive four important breeding males, with zookeepers, veterinarians, and conservation staff meeting TNR staff with the wallabies late the following night. In an example of the non-stop nature of the work undertaken by many partners at this time, the call for evacuation came on a Sunday afternoon, with all Victorian and ACT permits for the wallabies to cross state borders in place by Monday morning. In all, two wallabies were evacuated to Taronga Zoo, 17 to Mt Rothwell, and four to Healesville Sanctuary, thus highlighting the willingness of zoos and sanctuaries to assist each other at such times. Thankfully, TNR was not burnt in the fires. The wallabies stayed at Healesville Sanctuary for five weeks until they were able to be transferred safely back home.

### 2.4. Theme 7—Nature-Led Community Recovery

Catastrophic events, such as bushfires, can have a significant impact on people’s relationship with the natural environment. Applying psychological principles of working with trauma-affected communities, in conjunction with biodiversity recovery work, is an effective way to develop a paired-recovery feedback loop, in which evidence and stories about natural recovery support human recovery [28]. Zoos Victoria has linked with the ARI (see Hames [22]) to co-lead a nature-based Community Recovery Theme and has received support from an extended cohort of government agencies and NGOs. This program aims to enable social, economic, and environmental recovery of fire-impacted communities, while assisting the recovery of nature. Fire-impacted communities are supported to learn about how nature is responding and to design and deliver projects that benefit local plants, wildlife, and habitats. Participating in these projects, witnessing natural recovery, and gaining a sense of hope can actively support the recovery of fire-affected communities. The community recovery theme includes a small grants program for fire-affected communities, initially funded by Zoos Victoria and administered by the newly created Victorian government agency, Bushfire Recovery Victoria (BRV) [29]. It will provide access to biologists and ecological experts for advice and guidance, develop citizen science platforms for suitable projects, and elicit stories of shared recovery through multi-media storytelling opportunities. An evaluation of this novel program is being implemented.

Zoos Victoria has also developed a community-wide ‘Summer for Wildlife’ campaign to help Victorians understand how they might assist wildlife conservation and welfare during and after fires, heat wave events, and other summertime emergencies, including how to avoid animal collisions and assist wildlife on roads [30]. Such activities service the goal in Victoria’s Biodiversity 2037 Plan of ‘Victorians Value Nature’ [31], to increase the number of Victorians connecting with nature and acting to protect or enhance biodiversity. This work also aligns with modern zoo goals of connecting people with nature. There is increased focus across many zoos around the world to employ behavioural science methods to drive wildlife-friendly behaviours in zoo visitors and surrounding communities. It recognises the inextricable link between human wellbeing and environmental wellbeing.

### 2.5. Public Communications and Zoos Victoria’s Bushfire Emergency Wildlife Fund

During and after the fires, effective communication and transparency with media and the public were crucial. There was huge global interest in the Black Summer fires, the plight of Australia’s biodiversity, and response to injured and displaced wildlife. In particular, koalas became the face of the disaster, with offers of help arriving from around the world. In the first 10 weeks of 2020, the Zoos Victoria media team produced 3200 separate media stories across TV, radio, and print and online media. This helped to connect people with the koalas, Zoos Victoria’s Fighting Extinction species, and human communities impacted by the fires. Social media allowed proactive dissemination of information regarding the bushfires and our triage work, while also providing a platform to respond to enquiries from concerned and often distressed members of the public. Media ranged from articles on biodiversity loss and increasing threats to endangered species, to personal stories of koalas evacuated by military aircraft for emergency care following the devastating fires near Mallacoota and Croajingolong National Park, Victoria. We also published articles to assist communities in helping, rather than harming, native wildlife after fires [32].

Zoos Victoria seconded additional staff to answer phone calls as an influx of assistance was offered. However, we can only use specialised assistance and equipment for wildlife emergencies. In the case of an emergency with the magnitude of the Black Summer, the most useful offer was of funds to support immediate and long-term wildlife care and recovery. In early January 2020, Zoos Victoria provided a means for the community to assist during the disaster, through the establishment of the Bushfire Emergency Wildlife Fund (BEW Fund) [33]. This generated much needed funds to assist the immediate needs of wildlife triage, longer-term veterinary and rehabilitation care leading to animal releases back to the wild, as well as the ongoing longer-term recovery work required for threatened species. Importantly, these funds were quarantined to assist bushfire-affected wildlife and communities, and are available over the coming years. Many other external grants and funds available to assist wildlife come with a condition of spending all money within the following financial year (by June 2021). However, the recovery of species from catastrophic events is akin to a marathon, rather than a sprint—funds for recovery from the Black Summer will be required for decades.

Money was generously donated from a variety of sectors, including state, territory and Commonwealth governments; international zoos and wildlife agencies; philanthropic groups; Zoos Victoria members; and the public. In one notable case, Prague Zoo raised significant funds from their community in its appeal to assist Australia’s wildlife, even sending a delegation of staff members to fire-affected communities in early February 2020 to see how they could best help. Prague Zoo is now supporting key work to assist the recovery of a number of frog species and brush-tailed rock wallabies at Zoos Victoria. Schoolchildren set up lemonade stalls outside Healesville Sanctuary to raise funds and others emptied their piggy banks at our front desks. The outpouring of support from Victorian, Australian, and global communities was important not only to the fire fund, but to the staff who were working tirelessly across the fires—a symbol that we were not in this on our own, but had a global community backing our efforts.

The Zoos Victoria Customer Engagement Team (CET) provided a crucial service, fielding enquiries from a distressed Victorian community wanting to help the wildlife crisis. In January alone, the CET answered over 15,000 calls, of which 35% related to the BEW Fund. The CET was the first point of contact for often emotional and distressed callers. Staff provided a sympathetic ear for what had happened to callers directly, and how their families were coping throughout the crisis. At the end of each shift, team members shared the powerful and sometimes graphic stories they had heard and debriefed about the conversations. This allowed team members to best support one another, as well as the distressed community. During and after the Black Summer, our staff members were offered post-event debriefs and ongoing counselling support.

We developed a Zoos Victoria Response and Recovery Plan (ZVRRP) to ensure that Zoos Victoria use the funding entrusted to our organisation wisely and to communicate how we will spend the funds. The ZVRRP, prepared as a prospectus, is designed to nest within the larger Victorian BBRRP [15]. Our plan, in part, draws on the current five-year Wildlife Conservation Master Plan 2019–2024 [6], which outlines key actions required to prevent 27 highly endangered Victorian species from becoming extinct. Currently, the response plan includes the sections outlined below.

#### 2.5.1. Future-Proofing Wildlife Health and Welfare

This involves the employment of an emergency management specialist, the up-skilling of veterinary professionals in the assessment and care of bushfire-affected wildlife, the purchase of specialist equipment and machinery, additional veterinary costs, and building additional facilities to hold evacuated populations of native wild species from threatened habitats.

#### 2.5.2. Threatened Species Recovery

This is for projects specifically related to threatened species within bushfire areas, in addition to the 35 threatened species already on Zoos Victoria’s Fighting Extinction and Watch Lists.

#### 2.5.3. Nature-Based Community Recovery

This will assist in rebuilding local communities devastated by the bushfires, along with associated partners, as part of a larger recovery effort.

In addition, a number of NGO and not-for-profit organisations, including the International Fund for Animal Welfare (IFAW), Wildlife Victoria, and the Victorian Branch of the RSPCA, are supporting and partnering with the Zoos Victoria recovery efforts.

## 3. After the Fires: Planning and Future Resilience

Emergency events, such as the Black Summer fires, are predicted to occur at a higher frequency and greater intensity, and may overlap in the future due to climate change [5]. Thus, a key focus of Zoos Victoria’s bushfire emergency management work has been to enhance preparedness for this future. Determining the status and support required for threatened species and vulnerable ecosystems, the welfare of individual animals, and the nature-based recovery of human populations impacted by catastrophes are critical to gain resilience and have procedures, staff, and equipment in place for future emergencies. Such preparedness was highlighted in an Australia Royal Commission report (see below), which called upon state and territory governments working with relevant NGOs to establish best practice arrangements and responses relevant to emergency wildlife response and recovery.

### 3.1. Australian Royal Commission into National Natural Disaster Arrangements

The Australian Royal Commission into National Natural Disaster Arrangements (The Commission) [11] was established in February 2020 in response to the extreme bushfires of 2019–2020. It examined coordination, preparedness, response to, and recovery from disasters, as well as improving resilience and adaptation to changing climatic conditions and mitigating the impacts of such catastrophic events. The commission delivered its final report, outlining 80 recommendations, in October 2020, with the Commonwealth government’s response delivered the following month [34].

Briefly, the report noted Australia’s extraordinary biodiversity, which at an estimated 620,000 species represents between 7 and 10% of all species on earth. The majority of these species and ecological communities are unique to Australia. The commission noted the significant efforts to rescue and protect wildlife, ecosystems, and heritage sites during and since the 2019–2020 bushfires, with efforts relying on expert advice, data- and information-sharing, and fundraising efforts across individuals, communities, not-for-profit organisations, government agencies, environmental experts, and the private sector. It noted that some species that were not previously on threatened species lists may require inclusion in the threatened category. Zoos Victoria is working to determine whether any species require addition to its priority Fighting Extinction list for conservation action.

The Commission also noted there is a need to better integrate consideration of environment, wildlife, and heritage assets in emergency planning and response, including accessible data, such as on the location of environmental sites and the distribution of species and ecological communities, as well as priorities to guide response efforts. This includes state and territory governments working with relevant NGOs to establish best practice arrangements and responses relevant to emergency wildlife response and recovery. Effective wildlife response and recovery capabilities should be developed and integrated into emergency planning processes for disasters. The commission identified that “the development of clear and consistent national guidance on rescue and treatment of wildlife would support a coordinated approach to recovery”. Unfortunately, there are currently no agreed national standards for rehabilitation, assessment, treatment, and care for wildlife. Zoos Victoria, along with other zoos and stakeholders, are working with government agencies to develop such standards that could be used to assist wildlife in future emergencies across Australia and overseas.

The commission’s report recommended that Australian state and territory governments should ensure greater consistency and collaboration in the collation, storage, access, and provision of data on the distribution and conservation status of Australian flora and fauna. In response to the 2020 Report, the Commonwealth government, with all states and territories, has agreed to establish a uniform method for the assessment and listing of threatened species, which remains scientifically robust for individual species, while promoting a more consistent, efficient, and harmonious process.

### 3.2. Zoos Victoria’s Fighting Extinction Species

In 2009, Zoos Victoria commenced its Fighting Extinction program, which plays a leading role in the research, funding, public awareness, and recovery of 27 native priority species at risk of extinction (Fighting Extinction species), plus an additional eight threatened species on a Watch List for conservation action. Of these 35 species, 15 had habitat significantly affected by the fires, with some having over 80% of their remaining habitat burnt [15,35]. Surveys by Zoos Victoria and its partners are still underway to determine the extent of damage to habitat and the impact on the survival of species. Funds have been prioritised for actions for these species, including in situ protection and increasing or commencing ex situ programs where required. Affected species include those outlined below.

Frogs: Most of the known range of the northern and southern Corroboree frogs (*Pseudophryne corroboree* and *P. pengilleyi*) in NSW was burnt, as were large areas of habitat for the giant burrowing frog (*Heleioporus australiacus*), large brown tree frog (*Litoria littlejohni*), and spotted tree frog (*L. spenceri*). Most of the known range of the alpine tree frog (*L. verreauxii alpina*) and Martin’s toadlet (*Uperoleia martini*) were also burnt.

Reptiles: Large areas of habitat for the alpine she-oak skink (*Cyclodomorphus praeltus*) were burnt. The Guthega skink’s (*Liopholis guthega*) habitat in NSW was also affected, although the extent is unknown.

Birds: Two major breeding sites for the regent honeyeater (*Anthochaera phrygia*) in NSW were burnt. Areas of foraging habitat for the swift parrot (*Lathamus discolor*) on the NSW east coast were also burnt, although the impacts are as yet unclear.

Mammals: The entire extent of the brush-tailed rock wallaby’s core Victorian habitat (Snowy River National Park) was within the fire footprint, however the gorge itself escaped being burnt (also see above for the evacuation of wallabies from TNR). Victorian populations of the MPP have largely escaped fire impacts, however the populations in NSW have been heavily impacted (see above for supplementary feeding of possums after fires). Most historical sites of the smoky mouse in East Gippsland have been burnt, as well as current areas throughout the Victorian highlands. In NSW, 95% of the species’ remaining habitat was burnt. Large areas of the broad-toothed rat’s (*Mastacomys fuscus*) habitat have also been burnt, although the extent is still being investigated. Between 30 and 50% of the modelled habitat for the New Holland mouse was impacted by fires, with major damage occurring in NSW.

Invertebrates: The only known Victorian populations of the Key’s matchstick grasshopper (*Keyacris scurra*) are near Omeo, and it is not yet known whether nearby fires affected the habitat or population.

A review is underway to determine whether other species not currently on the Fighting Extinction or Watch List need to be added for priority protection and recovery action following the fires.

### 3.3. Work Underway for Future Resilience, Welfare, and Recovery

Key work underway through the Zoos Victoria BEW Fund [33] includes the initiatives listed below.

#### 3.3.1. Improved Capacity to Respond to Wildlife Welfare in Emergencies and Catastrophic Events

This includes emergency response training for veterinarians, veterinary nurses, and registered wildlife rehabilitators and rescuers, including field assessment of injured wildlife on fire grounds, in collaboration with DELWP. Training will be provided in the assessment and clinical care of wildlife at triage units. These efforts will help develop skills in the assessment and care of fire-affected wildlife; a wider pool of well-prepared veterinarians, veterinary nurses, and wildlife responders; and a database of trained and accredited first responders to assist native animals in future emergency events. Zoos Victoria is also conducting a systematic review of veterinary records from wildlife assessed and treated by veterinarians during the Black Summer fires. This will provide better understanding of commonly seen fire-related injuries, improved prognostic decision-making for rehabilitation or euthanasia, and assist in rapid assessment and management of fire-impacted wildlife presenting to triage units.

#### 3.3.2. Development of a Zoos Victoria Emergency Management Plan That Integrates with the State Emergency Management Plan

Zoos Victoria will provide capacity-building programs for our staff in emergency and crisis management to ensure the organisation is structured and prepared to respond to emergencies as part of a state-led response. The plan includes supporting mental health and well-being of staff involved in emergency responses, as working under demanding and stressful conditions, especially with people and animals under stress or in pain, takes its toll. Pre-season briefings; activation and deployment support; and post-emergency debriefings, counselling; and ongoing medium- and long-term psychosocial and wellbeing support will provide Zoos Victoria staff with best practice mental health care. The plan aims to strengthen existing stakeholder relationships within the emergency management and wildlife volunteer sectors to better embed collaborative best practice responses to wildlife needs in emergencies.

#### 3.3.3. Monitoring the Health and Welfare of Rehabilitated Koalas Following Release to the Wild

This ensures that veterinary management and rehabilitation protocols result in the best possible outcomes for fire-affected wildlife. We are monitoring 14 koalas, released after intensive care (see above), via GPS and radiotelemetry. The koalas receive pre- and post-release health assessments for us to better understand the welfare implications of release back to the wild of fire-affected wildlife. This project, implemented in partnership with DELWP, Parks Victoria, Federation University, and EcoPlan Australia and with funding from the IFAW, is particularly important when habitat has been badly impacted by fires and wildlife are released back to regenerating ecosystems. We will use information gained from this project to reassess pre-release rehabilitation and health assessment protocols, as well as release location selection criteria to ensure future rehabilitation and release efforts are targeted to result in the best possible welfare outcomes for koalas and other wildlife.

#### 3.3.4. Improved Wildlife Veterinary Care Capacity, Emergency Enclosures, and Transport Boxes

This work will ensure preparedness for future emergencies and includes the development of new multi-species hospital treatment and pre-release rehabilitation enclosures at Healesville Sanctuary; enhanced intensive care treatment facilities for wildlife at Melbourne Zoo; and in partnership with RSPCA Victoria, a koala hospital at Werribee Open Range Zoo. We are building increased surge capacity into Zoos Victoria’s three veterinary departments, with a wildlife response coordinator role proactively in place for rostering and planning the complicated logistics involved in veterinary emergency responses. Zoos Victoria is designing and building customized transport boxes for species likely to require emergency rescue in the future, along with storage facilities for transport boxes and catching equipment, such as traps and nets.

#### 3.3.5. Advanced Holding Facilities

Emergency facilities are being constructed for small mammals, amphibians, and birds in case of the need for extraction, with allowance for specific housing and temperature requirements for alpine species. We are designing and building mobile and flexible holding pens to allow for a range of emergency housing. Emergency enclosures for large species such as brush-tailed rock wallabies are under construction at Healesville Sanctuary. While enclosure preparations are focused on the 35 Fighting Extinction and Watch List species, there may be capacity for species with similar housing requirements to be included, depending on the bushfire threat and needs as requested by DELWP, Commonwealth and State Recovery Teams, and partners.

#### 3.3.6. Conservation Breeding Programs

Programs for the spotted tree frog are being expanded via new facilities, program coordinators, and specialist zookeepers at Healesville Sanctuary and Melbourne Zoo, with plans in place for giant burrowing frogs and large brown tree frogs. We are developing new species-specific facilities to enhance the MPP program at Healesville Sanctuary and commencing new captive programs for the New Holland mouse, smoky mouse, and broad-toothed rat at Melbourne Zoo, where required, based on wild population survey results (see Section 3.2). Additional staff capacity is being included, with new facilities and programs to allow the greatest focus on research and care of species. Field surveys and monitoring of fire-impacted invertebrates, frogs, reptiles, birds, and mammals are underway to determine the extent of fire impacts, status of species, key threats, and actions required for protection and recovery of species. Importantly, surveys are being performed in conjunction with government, university, museum, and other partners as a collaborative effort. At Zoos Victoria, new staff, including a native rodent biologist, mountain pygmy-possum field officer, broad-toothed rat field officer, and koala field officers, are being, or have been, employed. Additionally, we are working with a new brush-tailed rock wallaby master’s student from Federation University. We are also developing new techniques in advance, such as using Zoos Victoria’s conservation detection dogs to find surviving broad-toothed rats, in order to assist future rescue efforts.

## 4. Conclusions

Zoos Victoria’s response to the Black Summer was part of a larger effort coordinated by the Victorian government, made possible through multiple collaborative partnerships. The work outlined above has been possible due to coordinated and inclusive state-wide and national approaches to wildlife in emergency management. The devastating Black Summer fires highlighted the crucial nature of preparedness to assist with triage, ongoing veterinary care and rehabilitation, evacuation, monitoring after release to the wild, supplementary feeding, and other wildlife emergency responses. Expertise and forward planning, such as an active Zoos Victoria Wildlife Conservation Masterplan [6], triage experience, having translocation plans in place (such as for the eastern bristlebird), and key programs such as the development of nutritionally suitable supplementary food for wildlife (such as for the MPP) gave Zoos Victoria the ability to mobilise quickly in the face of unprecedented bushfires. The huge global focus from communities and media and the extraordinary offers of help and funding have far exceeded what we could have imagined. Increased staffing and support for staff were crucial during these times. Using the donated funds to prepare for future events via increased staffing and training, development of emergency protocols and plans, purchase of new equipment and enclosures, monitoring and recovery actions for fire-affected species, and supporting communities will allow us to assist in even greater ways in the future. As a modern zoo, we have shown the significant role that zoos and their specialist personnel can play in conservation and animal welfare during emergency events. We hope that outlining our work during and after the Black Summer will stimulate a focus on the critical wildlife response work possible by zoos. It will be a powerful movement if zoos harness their expertise and resources to support their local wildlife in need around the world.

Concern for wildlife welfare increased dramatically across the Black Summer, highlighting the value communities place on wildlife and the environment. Through generous donations to Zoos Victoria’s BEW Fund, we are implementing actions to best prepare for the future, assist wildlife welfare and triage, advance the recovery and resilience of threatened species, and aid community healing. Catastrophes, including bushfires, are increasing in magnitude, frequency, and intensity; as in all conservation programs, we can hope for the best, but must prepare for the worst.

## Figures and Tables

**Figure A1:**
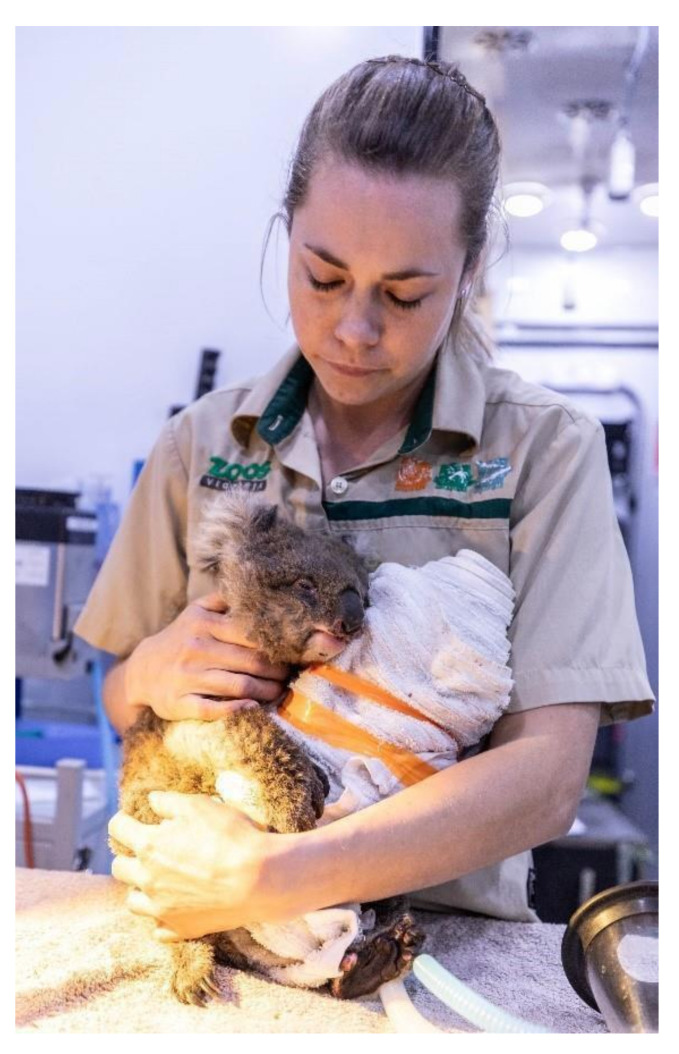
Koala assessment by Zoos Victoria staff (Marcia Reiderer).

**Figure A2:**
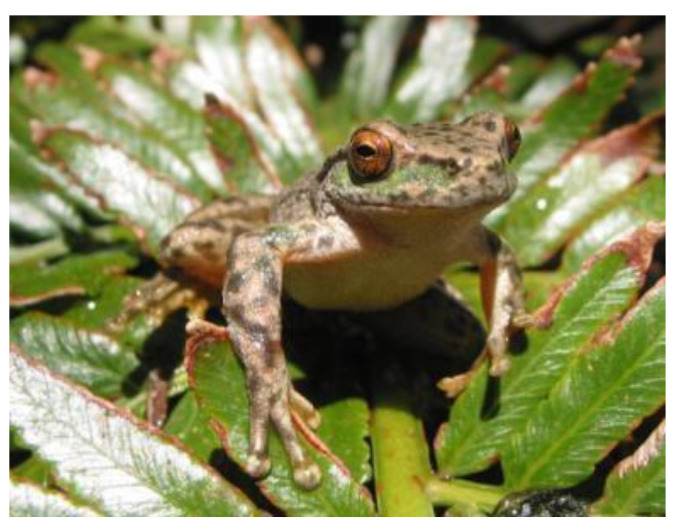
Spotted tree frog (Glen Johnson).

**Figure A3:**
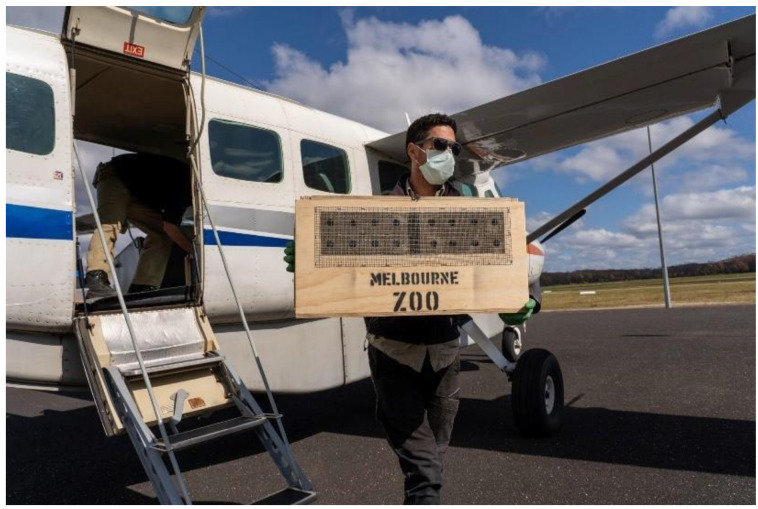
Eastern bristlebird extraction by Zoos Victoria staff (Darryl Whitaker).

## Data Availability

No new data were created or analyzed in this study. Data sharing is not applicable to this article.

## Appendix A

### Appendix A.1. DELWP Bushfire Biodiversity Response and Recovery Program—Themes and Focus Areas

Direct source: https://www.wildlife.vic.gov.au/home/biodiversity-bushfire-response and recovery (Accessed on 20 December 2020) [15].

### Appendix A.2. Bushfire Biodiversity Response and Recovery Actions

The Victorian government is providing $51.5 million for the Bushfire Biodiversity Response and Recovery program to support Victoria’s bushfire impacted wildlife and biodiversity. This is supported by $3 million from the Australian government’s wildlife and habitat bushfire recovery package. It delivers actions across a range of themes and focus areas.

### Appendix A.3. Theme 1—Immediate Reconnaissance

#### Appendix A.3.1. Funding Allocation: $2.1 Million (Approximately)

Assessing the specific impacts of the fires on species or locations of concern enables more targeted actions. This theme assesses the status of key plants, animals, and ecosystems following the fires to inform management actions. The focus is on species or ecosystems that have had all known populations or locations affected or where the level of impact is not fully known.

#### Appendix A.3.2. Key Actions

- Ground surveys and aerial surveys (where appropriate);
- Surveys following standard scientific methods to ensure comparisons with pre-fire surveys;
- Provision of artificial habitat features;
- Further risk assessment.

### Appendix A.4. Theme 2—Wildlife Health and Welfare

#### Appendix A.4.1. Funding Allocation: $1.0 Million (Approximately)

Responding to the welfare of wildlife injured during fire is a critical element of the Victorian government’s fire response procedures (Figure A1). There is also significant community concern for wildlife during fire events. This theme builds on existing arrangements and seeks to more closely engage with community on wildlife response during fires. This includes identifying improved coordination and communication with wildlife carers and advocacy groups.

#### Appendix A.4.2. Key Actions

- Working with key wildlife welfare organisations around improved engagement;
- Development of targeted training programs for veterinarians on wildlife assessment, burns treatment, and triage;
- Undertaking a post-rehabilitation study to track released koalas that were taken into care following the fires;
- Development of an electronic recording system for the collection of wildlife assessment and triage data.

### Appendix A.5. Theme 3—Emergency Extraction

#### Appendix A.5.1. Funding Allocation: $1.5 Million (Approximately)

The effects of the fires can impact the ability of some threatened species to persist in their natural environment. This theme prioritises options for the emergency extraction of these species at risk of extinction or further decline (Figure A2). The aim is to collect critically threatened fauna species and keep them in temporary care or at an alternative wild location, as well as to collect threatened flora seeds and cuttings for propagation, until the species can be returned to the wild (Figure A3).

#### Appendix A.5.2. Key Actions

- Emergency extraction and temporary housing or translocation of critical fauna (e.g., fish and aquatic invertebrates, eastern bristlebird);
- Collection of seed or plant materials and ex situ propagation for key plant species.

### Appendix A.6. Theme 4–Intensified and Sustained Treatment of Threats

#### Appendix A.6.1. Funding Allocation: $25.0 Million (Approximately)

Wildlife and habitats affected by fires are more vulnerable to introduced pest plants and animals. Some pests can thrive in a post-fire landscape, increasing their impact on an environment that is already compromised. This theme delivers an integrated and coordinated program focused on introduced herbivore and predator control, as well as weed control, to reduce the heightened risks in burnt and adjacent areas.

#### Appendix A.6.2. Key Actions

- Aerial shooting of introduced pest animals on public land;
- Targeted ground control of introduced pest animals;
- Targeted weed control.

### Appendix A.7. Theme 5

#### Appendix A.7.1. Reading and Healing Country–Funding Allocation: $2.1 Million (Approximately)

This theme will ‘heal Country through reading Country’. It will enable Traditional Owners to apply ecological knowledge and lead the coordination of cultural heritage and related activities in the fire-affected areas.

#### Appendix A.7.2. Landscape Resilience–Funding Allocation: $19.5 Million (Approximately)

The recovery of species affected by the fires will benefit from actions both within and beyond the 2019–20 fire area. This theme focuses on maximising the long-term, state-wide resilience of species and their habitats across the landscape, with emphasis on the species of greatest concern after fires.

#### Appendix A.7.3. Key Actions

- Create and support a safe haven network of ecological refuges across the state;
- Revegetation and reseeding;
- Spread the risk to a species from future disturbance events by establishing a new population;
- Spread the risk to a species from population failures through genetic mixing to improve the fitness of populations and manage threats;
- Applying a cultural landscape lens to species renewal and resilience, using cultural knowledge and practices.

### Appendix A.8. Theme 6—Knowledge, Data, and Preparedness

#### Appendix A.8.1. Funding Allocation: $1.8 Million (Approximately)

This theme will improve our knowledge of expected biodiversity outcomes from post-fire management of threats and will improve preparedness for protecting biodiversity during future fire events. This includes identifying priority options across Victorian landscapes to reduce the risks to biodiversity in the event of future fires.

#### Appendix A.8.2. Key Actions

- Developing strategic guidance to prioritise direct interventions in species populations (e.g., to address genetic risk);
- Better targeting of research and monitoring of management effectiveness;
- Continuous improvement of biodiversity information and decision-support tools;
- Developing systems to improve data flow across agencies and to the public.

### Appendix A.9. Theme 7—Nature-Led Community Recovery

#### Appendix A.9.1. Funding Allocation: $0.1 Million (Approximately)

Connecting with nature has benefits for people and for biodiversity. The aim of this theme is to enable the social, economic, and environmental recovery of fire-impacted communities, led by nature. It will support people’s recovery from the fires by facilitating their engagement in activities to assist the recovery of nature.

Fire-impacted communities will be supported to learn about how nature is responding and to design and deliver projects that benefit local plants, wildlife, and habitats. Participating in these projects, witnessing natural recovery, and gaining a sense of hope and agency can actively support the recovery of fire-affected communities.

#### Appendix A.9.2. Key Actions

- Providing grants to support small community-led, place-based projects;
- Providing access to scientists or expertise to assist understanding and opportunity;
- Supporting meaningful citizen science and volunteering projects;
- Encouraging people to notice nature recovery;
- Sharing stories of recovery.
